# Electrochemical Determination of 4-Bromophenoxyacetic Acid Based on CeO_2_/eGr Composite

**DOI:** 10.3390/bios12090760

**Published:** 2022-09-15

**Authors:** Haijun Du, Yan Zhang, Xin Wang, Huali Hu, Jixing Ai, Huanxi Zhou, Xia Yan, Yang Yang, Zhiwei Lu

**Affiliations:** 1 School of Chemical Engineering, Guizhou Minzu University, Guiyang 550025, China; 2 School of Materials Science and Engineering, Guizhou Minzu University, Guiyang 550025, China; 3 School of Mechatronics Engineering, Guizhou Minzu University, Guiyang 550025, China; 4 College of Science, Sichuan Agricultural University, Ya’an 625014, China

**Keywords:** plant growth regulators, 4-bromophenoxyacetic acid, electrochemical determination, lowest detection limit, CeO_2_/eGr composite

## Abstract

The determination of plant growth regulators is of great importance for the quality monitoring of crops. In this work, 4-bromophenoxyacetic acid (4-BPA), one of the phenoxyacetic acids, was detected via the electrochemical method for the first time. A CeO_2_-decorated electrochemical exfoliated graphene (eGr) composite (CeO_2_/eGr) was constructed as the sensor for sensitive detection of 4-BPA due to the synergistic effect of the excellent catalytic active sites of CeO_2_ and good electron transference of the eGr. The developed CeO_2_/eGr sensor displayed a good linearity in a wide range from 0.3 to 150 μmol/L and the lowest detection limit of 0.06 μmol/L for 4-BPA detection. Electrochemical oxidation of 4-BPA follows a mix-controlled process on the CeO_2_/eGr electrode, which involves 2e in the transference process. This developed CeO_2_/eGr sensor has excellent repeatability with a relative standard deviation (RSD) of 2.35% in 10 continuous measurements. Moreover, the practical application of the sensor for 4-BPA detection in apple juice has recoveries in the range of 90–108%. This proposed CeO_2_/eGr sensor has great potential for detecting plant growth regulators in the agricultural industry.

## 1. Introduction

Plant growth regulators (PGRs) are widely used to promote a crop’s productivity and quality [1,2], control crop type [3], resist biotic and abiotic stress [4], regulate differentiation of cells, control weeds [5], and for phytoremediation [6]. 4-bromophenoxyacetic acid (4-BPA) is a PGR that can control weeds, accelerate plant growth, and enhance the fruit setting rate. However, inappropriate usage of 4-BPA will cause malformations and affect the quality of the crop. Furthermore, accumulation in the crop can be a detriment to other plants, animals, and to human health. Therefore, it is of great necessity to develop convenient, sensitive, and reliable analytical methods for 4-BPA determination. Unfortunately, to the authors’ knowledge, only Sutcharitchan [7] has developed a liquid chromatography-tandem mass spectrometry (LC-MS) method for 4-BPA determination in Chinese herbs.

4-BPA is a phenoxyacetic acid, and a variety of analytical methods for phenoxyacetic acid detection have been developed, including capillary electrophoresis with laser-induced fluorescence [8], ultra-high liquid chromatography-mass spectrometry [9], headspace gas chromatography high-performance liquid chromatography [10] and electrochemical methods [11,12,13,14,15]. Compared to these methods, electrochemical methods have the advantage of high sensitivity, low cost, portability, and simple operation [16,17]. The direct interaction between the electrode surface and analyte can reduce the lowest limitation and improve the detection range, which has the potential to establish a rapid on-site inspection method. In addition, the advantages of the electrochemical sensors include fast response, on-site deployment, effective detection without sample pretreatment and the modified nanocomposite exhibits high electrocatalytic activity for an analyte [18]. Because of its electrochemical activity, 4-BPA is detectable via electrochemical methods.

Ceria (CeO_2_) is an n-type semiconductor that has the potential to be used as a sensing material, since it has excellent redox characteristics and is highly catalytic, biocompatible, and non-toxic [19,20]. Moreover, CeO_2_ can selectively bind with organic molecules due to its characteristics of oxygen vacancies, free electrons, and high chemical stability [21]. However, it is hard to directly use as modifier material in electrochemical sensors as CeO_2_ suffers from poor conductivity and easy aggregation. Therefore, it is essential to enhance its sensing activities by introducing conductive support.

Graphene (Gr) is one of the best candidates for the construction of electrochemical sensors, as it has unique and extraordinary physical and chemical properties, such as high specific surface area, low charge-transfer resistance, excellent electrochemical activity and high electrical conductivity [22,23], which improve the value of heterogeneous electron transfer (HEF) and the output signal intensity [24,25]. Over the past few decades, different protocols, including top-down approach (e.g., chemical vapor deposition) and bottom-up methods (e.g., electrochemical exfoliation) have been developed [26,27]. Among these methods, electrochemical exfoliation of graphite has the advantages of being low-cost and environmentally friendly, easily made and scalable, and the electrochemical conditions are controllable. Moreover, the exfoliated graphene is a zero-gap semiconductor; it can be doped with p-block elements (N, S, P, B, and metal oxides) and d- block elements (inherent impurities) [26,28] to improve the HEF to promote the interaction between target molecules and the electrode. In the study of Liu [29], gold-palladium nanoparticles were cast on the graphene nano-platelets, and the nanocomposites showed high electrocatalytic ability towards the oxidation of hydrazine. Li [30] synthesized three kinds of CeO_2_ nanostructures and then loaded them on the graphene nanoplatelets to detect phenolic pollutants, which exhibited excellent electrochemical activity. Previously, our group has developed an electrochemical exfoliation graphene (eGr) sensor, which displayed the lowest detection limit (LOD) of 0.15 µM for electrochemical determination of Kinetin [31]. Therefore, CeO_2_ was decorated on the surface of eGr to form a nanocomposite that will have a synergetic effect between eGr and CeO_2_, thus forming a sensitive, selective and promising electrode system for 4-BPA detection. In the electrochemical determination process, several types of electrodes are suitable for electroanalytical applications, such as glassy carbon electrodes (GCE), gold electrodes (GE), carbon paste electrodes (CPE) and pencil electrodes. Among these electrodes, GCE shows attractive electrochemical reactivity, negligible porosity, good mechanical rigidity and good repeatability and reproducibility [32].

Herein, CeO_2_ nanocubes were synthesized via the hydrothermal method and electrochemical exfoliated graphene was prepared through the previous method [25]. A CeO_2_ decorated eGr composite was constructed and employed to detect 4-BPA for the first time. This developed eGr/CeO_2_ sensor has a linear range from 0.3 to 150 µM and the LOD of 0.06 µM for 4-BPA determination, which shows great potential for the detection of plant regulators in the agricultural industry.

## 2. Materials and Methods

### 2.1. Reagents and Materials

All reagents used in the experiments are analytical reagent grade and without any treatment. Graphite sheets were purchased from the local electronic market. Ce(NO_3_)_3_∙6H_2_O, 4-BPA, indole 3-acetic acid, naphthalene acetic acid and 6-benzylaminopurine were purchased from Macklin biochemical Technology Co., Ltd. (Shanghai, China). A solution of 0.1 M 4-BPA was prepared by dissolving a suitable amount of 4-BPA in alcohol and diluting the mixture to 10 mL, then the solution was stored in a refrigerator at 4 °C. Phosphate buffer solution (PBS) was used as a supporting electrolyte by combining a stock solution of 0.1 M KH_2_PO_4_ (Aladdin Reagent Co., Ltd., Shanghai, China) and 0.1 M NaH_2_PO_4_ (Aladdin Reagent Co., Ltd., Shanghai, China), then 0.1 M H_3_PO_4_ (Aladdin Reagent Co., Ltd., Shanghai, China) and 0.1 M NaOH (Sinopharm Group Chemical Reagent Co., Ltd., Shanghai, China) were, respectively, used to adjust the pH to the desired value.

### 2.2. Preparation of CeO_2_, eGr, and eGr/CeO_2_ Composites

CeO_2_ nanocubes were prepared using the hydrothermal method. Firstly, 0.6948 g Ce(NO_3_)_3_∙6H_2_O and 0.0224 g hexamethylenetetramine (HMT) was dissolved in 40 mL distilled water and 40 mL ethanol. The resulting solution was vigorously stirred for 20 min at room temperature, then it was transferred into a 100 mL Teflon-lined stainless-steel autoclave, and hydrothermally heated at 180 °C for 20 h. After that, the product was collected by centrifuging, and alternatively washed with distilled water and ethanol to a neutral pH, and then dried in an oven at 80 °C for 12 h. Finally, the obtained yellow powder was calcined at 400 °C for 5 h.

For eGr preparation, a two-electrode cell was used including graphite foil as an anode, a platinum net as a cathode, and 0.1 M (NH_4_)_2_SO_4_ as a supporting electrolyte, whereas the eGr was obtained with the aid of SO_4_^2−^ intercalation and oxidization to produce sulfur dioxide and oxygen gases, then the product was centrifuged, filtrated and dried overnight.

Next, 9 mg CeO_2_ and/or 9 mg eGr were dispersed in 9 mL *N, N*′-dimethylformamide (DMF) solution with vigorous stirring and then ultra-sonicating for 2 h to obtain the CeO_2_, eGr, and CeO_2_/eGr suspensions. The glassy carbon electrodes (GCEs) were polished to a mirror-like surface with 0.3 and 0.5 µm Al_2_O_3_ slurries on chamois leather, then alternately rinsing with distilled water/ethanol (1:1, *v*/*v*) solution and double distilled water for 3 min. Finally, CeO_2_, eGr, and CeO_2_/eGr suspensions were, respectively, drop-coated on the mirror-like surface of GCEs to obtain CeO_2_/GCE, eGr/GCE, and CeO_2_/eGr/GCE electrodes.

### 2.3. Characterization

Scanning electron microscope (SEM, Thermo scientific Apreo 2C, Waltham, MA, USA) and transmission electron microscope (TEM, FEI Tecnai F20, Hillsboro, OR, USA) and High-resolution transmission electron microscope (HRTEM, FEI Tecnai F20, Hillsboro, OR, USA) were used to analyze the surface morphologies of CeO_2_, eGr and CeO_2_/eGr nanocomposite. Raman spectra were collected with a 532 nm diode laser by Thermo Fisher Dxr2xi (Waltham, MA, USA). X-ray diffraction (XRD) patterns were recorded by the PANayltical Empyrean system (Almelo, The Netherlands). X-ray photoelectron spectra (XPS) were recorded by Thermo Scientific K-Alpha (Waltham, MA, USA).

Cyclic voltammetry (CV), linear sweep voltammetry (LSV), and differential pulse voltammetry (DPV) measurements were performed on a CHI 660E electrochemical workstation (CH Instruments ins., Shanghai, China) and a classic three-electrode system. CVs were carried out with the potential range from 0.8 to 1.5 V at a scan rate of 0.1 V s^−1^. LSVs were carried out with the potential range from 0.9 to 1.6 V at a scan rate of 0.1 V s^−1^. DPVs were carried out from 0.8 to 1.5 V with parameters of 0.05 V amplitude, 0.06 s pulse width, 0.02 s sampling width, 0.5 s pulse period and 30 s rest time. A glassy carbon electrode (GCE, diameter 3 mm) or modified GCE as the working electrode, a platinum wire served as a counter electrode and Ag/AgCl was used as the reference electrode.

### 2.4. Sample Pretreatment

The purchased apple was squeezed into juice (120 g) and the fresh apple juice was mixed with distilled water and sonicated for 20 min (at room temperature), then centrifuged for 5 min with 10,000 r/min to obtain the supernatants. Supernatants were collected for further quantification of 4-BPA.

## 3. Results and Discussion

### 3.1. Morphology and Phase Structure Characterization

**Figure 1:**
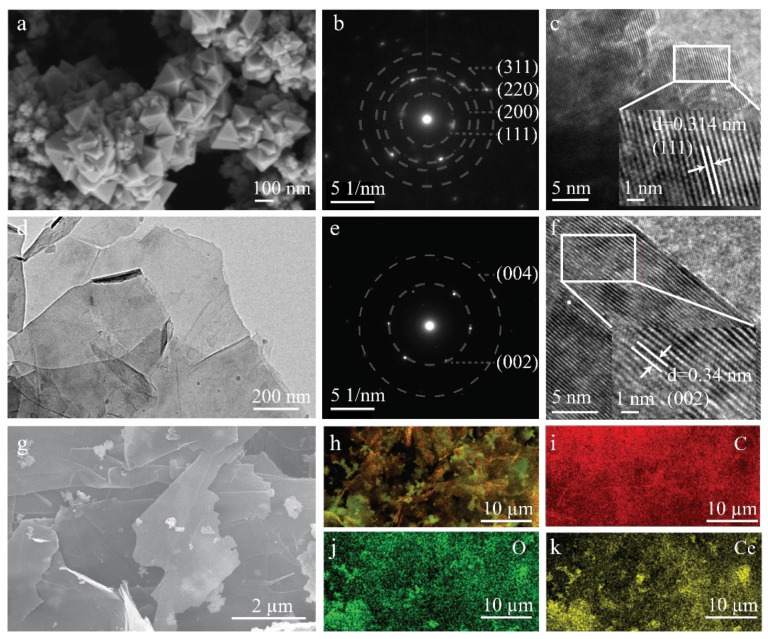
SEM image (**a**), SEAD (**b**) and HRTEM (**c**) of CeO_2_, TEM (**d**), SEAD (**e**) and HRTEM (**f**) of eGr, SEM image (**g**) and corresponding elemental mapping of the selected area (**h**), C (**i**), O (**j**) and Ce (**k**) of CeO_2_/eGr.

The morphology of the synthesized CeO_2_ was observed by SEM, which presents a nano-cubic structure, and agglomerated together due to the nanosize effect (Figure 1a). The diffraction rings in the selected area electron diffraction (SAED) suggest the as-synthesized CeO_2_ is polycrystalline and mainly exists in (111), (200), (220), and (311) crystallite planes (Figure 1b). From HRTEM (Figure 1c), there is a main lattice space distance of 0.314 nm which belongs to (111) crystallite plane of CeO_2_. For the eGr sample, the TEM image indicates the prepared eGr displays a layered structure (Figure 1d), and the SAED indicates that the eGr mainly presents (002), and (004) crystallite planes (Figure 1e), the existed lattice space distance of 0.34 nm matches well with the theoretical value of graphene (002) crystallite plane (Figure 1f); it verifies the high quality of the prepared eGr. The CeO_2_/eGr nanocomposite used here was 1:1 in a weight ratio (1:1 wt.). Figure 1g shows that CeO_2_ is uniformly loaded on the surface of eGr and forms on the selected area (Figure 1h); the corresponding elemental mapping illustrates that the elements of C, O and Ce exist in the CeO_2_/eGr nanocomposite (Figure 1i–k).

**Figure 2:**
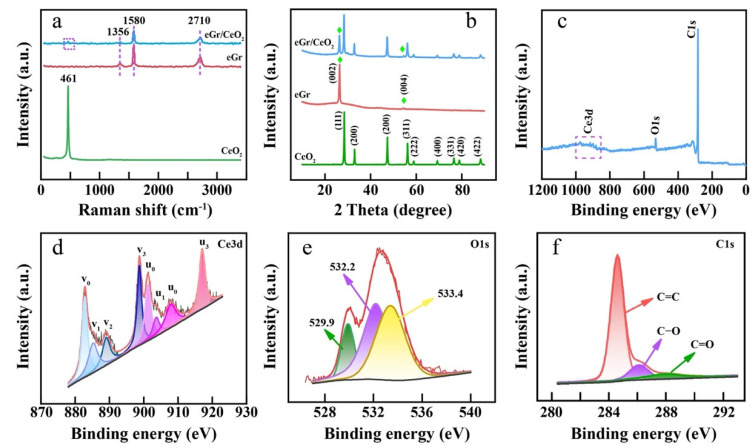
(**a**) Raman spectra, (**b**) XRD patterns of eGr, CeO_2_, and CeO_2_/eGr, (**c**) XPS survey spectrum, (**d**) Ce3d, (**e**) O1s and (**f**) C1s spectra of CeO_2_/eGr composite, respectively.

The crystallite structure and composition of CeO_2_, eGr and CeO_2_/eGr composite (1:1 wt.) were evaluated by Raman spectra, XRD and XPS. In Raman spectra of Figure 2a, it shows a characteristic peak located at 461 cm^−1^ for the CeO_2_ sample, which stems from the symmetrical stretching of Ce-O vibrational and originates from the F2g vibrational mode of the CeO_2_ phase [33]. For eGr, the peaks at 1356, 1580 and 2710 cm^−1^ were assigned to D, G and 2G bands of graphene, respectively [34]. The D band at ~1356 cm^−1^ is derived from the defects and structural disorder in the sp^2^-carbon nanomaterials. The G band at 1580 cm^−1^ is related to the in-plane vibrations of the 2D hexagonal graphene lattice. The CeO_2_/eGr composite sample possesses both Raman characteristics of CeO_2_ and eGr. XRD was used to analyze the structure of the prepared materials. In Figure 2b, the strong and sharp diffraction peaks indicate all the samples are in good crystallinity. For eGr, two diffraction peaks at 26.4°and 54.5° are observed, which are related to (002) and (004) planes of graphene, this is in accordance with the SEAD result (Figure 1e). For CeO_2_, the diffraction peaks located at 28.5°, 33.1°, 47.5°, 56.3°, 59.1°, 69.4°, 76.7°, 79.1°and 88.4° correspond to (111), (200), (220), (311), (222), (400), (331), (420) and (422) planes (JCPDS 81-0792). The CeO_2_/eGr composite also contains all the characteristic peaks of CeO_2_ and eGr. Based on the CeO_2_/eGr composite containing all the features of CeO_2_ and eGr, the chemical composition of the CeO_2_/eGr composite was further characterized by XPS. The XPS survey spectrum (Figure 2c) reveals the existence of Ce, O, and C elements in the CeO_2_/eGr composite. The Ce3d electron core line was analyzed and is depicted in Figure 2d; it can be deconvoluted into eight peaks and labeled as v_0_, v_1_, v_2_, v_3_ (3d_3/2_ region), and u_0_, u_1_, u_2_, u_3_ (3d_5/2_ region). Peaks v_0_, v_2_, v_3_ and u_0_, u_2_, u_3_ are characteristics of Ce(IV) 3D final states, while, v_1_ and u_1_ are Ce(III) 3D final states [35]. Therefore, the as-prepared CeO_2_ contains part of Ce(III), and the percentage of Ce(III) was calculated by Equation (1), which is based on the fitted areas of the corresponding peaks of Ce(III) and Ce(IV) [36].

(1) $${\left({\mathrm{Ce}}^{3+}\right)}_{\mathrm{surf}}=\frac{\mathrm{Ce}(\mathrm{III})}{\mathrm{Ce}(\mathrm{III})+\mathrm{Ce}(\mathrm{IV})}$$

The calculated percentage of Ce(III) is ~20%, which is similar to the previously reported CeO_2_ nanomaterials [36]. The presence of Ce(III) indicates the formation of oxygen vacancies, which can provide catalytically active sites for the sensor. The O1s spectrum can be separated into three peaks as illustrated in Figure 2e, the peak located at ~529.9 eV corresponds to the crystal lattice oxygen in CeO_2_. The peak located at 532.2 eV and 533.4 eV could be, respectively, related to the oxygen vacancies and the adsorbed oxygen on the composite [37,38]. The C1s spectrum can be separated into three peaks (Figure 2f). The peak placed at 284.6 eV corresponds to the sp2 carbon atoms or can be attributed to C=C [39]. The other small peaks at 286.1 and 287.9 eV correspond to C–O and C=O on the surface of the composite, respectively.

### 3.2. The Electrochemical Characteristic of the Prepared Electrode

**Figure 3:**
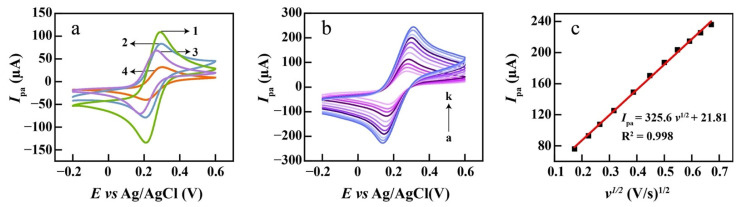
(**a**) CVs of CeO_2_/eGr/GCE in the presence of 5 mM [Fe(CN)6]^3−/4−^ solution in aqueous 0.1 M KCl. CeO_2_/eGr/GCE (1), eGr/GCE (2), CeO_2_/GCE (3), bare GCE (4); (**b**) CVs of CeO_2_/eGr/GCE in the presence of 5 mM [Fe(CN)6]^3−/4−^ solution in aqueous 0.1 M KCl at various scan rate (from a to k): 0.03, 0.05, 0.07, 0.1, 0.15, 0.2, 0.25, 0.3, 0.35, 0.4, 0.45 V s^−1^. (**c**) The plot of peak currents vs. *v*^1/2^.

The prepared eGr, CeO_2_, and CeO_2_/eGr composites (1:1 wt.) were, respectively, cast on a glassy carbon electrode (eGr/GCE, CeO_2_/GCE and CeO_2_/eGr/GCE), and their electrochemical performances were firstly estimated by Cyclic voltammetry (CV) at 50 mV s^−1^ in the solution of 5 mM [Fe(CN)_6_]^3−/4−^ and 0.1 M KCl. The bare GCE electrode was conducted as the control sample. From Figure 3a, all electrodes show different levels of electrochemical activity, after evaluating the redox peak current densities and CV curve area, the electrochemically active follows the order of CeO_2_/eGr/GCE > eGr/GCE > CeO_2_/GCE > GCE. This suggests that the CeO_2_/eGr/GCE has the largest specific surface area, and the best electrochemically active and kinetic, which could arise from the synergistic effects of excellent catalytic active sites of CeO_2_ and good electron transference of eGr. In addition, the standard heterogeneous rate constant (k^0^) for bare GCE, CeO_2_/eGr/GCE, eGr/GCE, CeO_2_/GCE were calculated by Nicholson’s equation [40] and the values are, respectively, 0.0041 cm·s^−1^, 0.0077 cm·s^−1^, 0.0045 cm·s^−1^, 0.0049 cm·s^−1^. The CeO_2_/eGr/GCE has the highest value of 0.0077 cm s^−1^ that verifies CeO_2_/eGr composite provides the best conditions for electron transfer.

The electroactive surface area is a critical factor for the electrochemical sensor, which was estimated in [Fe(CN)_6_]^3−/4−^ solution with scan rates ranging from 0.03 to 0.45 V s^−1^ via the Randles-Sevcik equation (Equation (2)) [41].

(2) $$I_{\mathrm{pa}}=(2.69\times 10^{5})n^{3/2}AD^{1/2}Cv^{1/2}$$

where *n* refers to electron transfer number, *A* is the active surface area, *C* is the concertation of [Fe(CN)_6_]^3−/4−^, *v* is the scan rate and *D* is the diffusion coefficient. Here, *n* = 1, D = 6.6 × 10^−6^ cm^2^ s^−1^ [34] for 5 mM K_3_[Fe(CN)_6_] solution containing 0.1 M KCl. For CeO_2_/eGr/GCE (Figure 3b), the active surface area of CeO_2_/eGr/GCE was calculated to be 0.097 cm^2^ (Figure 3c), which is higher than that of the electroactive surface areas of GCE (0.04 cm^2^), eGr/GCE (0.08 cm^2^), and CeO_2_/GCE (0.045 cm^2^), which is displayed in the Supporting Information, Figure S1. This result agrees well with the electrochemical activity order of the prepared sensors. The roughness factor (*f_r_*) of the electrochemical sensors was calculated to evaluate the actual active surface area by comparing the oxidation peak current (*I*_pa_) of the prepared sensor to bare GCE for [Fe(CN)_6_]^3−/4−^ reaction [42]. Peaks ratio is equal to areas ratio according to the proposed Equation (3) [42].

(3) $$f_{r}=\frac{I_{P2}}{I_{P1}}=\frac{A_{2}}{A_{1}}$$

The *f_r_* determined by electrochemical methods depends not only on the size of the electrode (the actual surface), but also on the number of redox centers that can be reached on the surface. Therefore, the *f_r_* was calculated to be 2.425, 2, and 1.625 for CeO_2_/eGr/GCE, eGr/GCE, and CeO_2_/GCE, respectively.

### 3.3. The Electrochemical Performance of the Prepared Electrode for 4-BPA Detection

**Figure 4:**
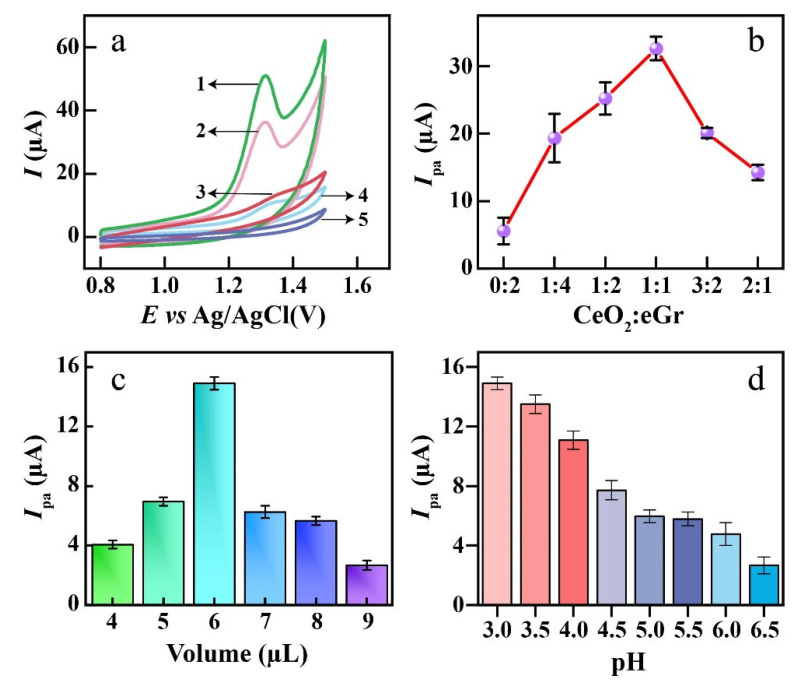
(**a**) CVs of CeO_2_/eGr/GCE (1), eGr/GCE (2), bare GCE (3) and CeO_2_/GCE (4) in 0.1 M PBS solution containing 50 µmol L^−1^ 4-BPA at the scan rate of 100 mV s^−1^, CeO_2_/eGr/GCE (5) in PBS solution without 4-BPA; (**b**) Influence of the ratio between CeO_2_ and eGr on the response of 4-BPA based on LSVs. (**c**) the response of 10 µmol L^−1^ 4-BPA to different volumes of composite nanomaterial in PBS solution (pH = 3). (**d**) Influence of pH on the peak current of 4-BPA in 0.1 M PBS solution.

The GCE, eGr/GCE, CeO_2_/GCE and CeO_2_/eGr/GCE (1:1 wt.) for 4-BPA detection were characterized by CV in the electrolyte with and without 50 µmol L^−1^ 4-BPA in 0.1 mol L^−1^ phosphate buffer (pH = 3). As displayed in Figure 4a, when the presence of 50 µmol L^−1^ 4-BPA, all electrodes present one oxidation peak, which indicates the 4-BPA is electrochemically detectable and the reaction of 4-BPA is irreversible. The CeO_2_/eGr/GCE (1:1 wt.) shows the highest oxidation peak current (*I*_pa_) and the lowest onset potential; this verified that the CeO_2_/eGr composite has the best sensitivity for electrochemical detection of 4-BPA, which should be attributed to the synergetic effect of the catalytic properties of CeO_2_ and the fast electron transference of eGr. The ratios between CeO_2_ and eGr have further been measured and shown in Figure 4b. With the CeO_2_:eGr ratio increasing from 0:4 to 1:1, the oxidation peak current of 4-BPA increases and reaches the maximum at the ratio of 1:1, then the peak current drops with the further increase in the CeO_2_ content. The reason could be that CeO_2_ is a semiconductor, and it provides electrocatalytic activity sites. When the CeO_2_ content is too low, it will not create enough activity sites. While the content is higher than 1:1, the conductivity and electron transference of the electrode will decrease. Therefore, the optimum ratio was 1:1 for 4-BPA detection and selected in the following study.

The different loading amounts of CeO_2_/eGr composite on GCE were measured with 10 µmol L^−1^ 4-BPA (Supporting information, Figure S2). It was found that the oxidation peak current of 4-BPA increases with the loading volume, increasing to 6 µL, while the response decreased when the loading amount further increased (Figure 4c). This could be due to the lack of cover on the electrode surface when the loading composites were below 6 µL, while too much loading amount would have hindered the activity sites that cause the decrease in the response peak current [43].

The PBS, Britton–Robison (B-R), and acetic acid-sodium acetate buffer solutions were evaluated as supporting electrolytes for 4-BPA detection (Supporting information, Figure S3). Among these supporting electrolytes, the PBS buffer solution shows more sensitivity for 4-BPA detection. Therefore, PBS was selected as a supporting electrolyte. Moreover, pH is another key impact factor for electrochemical analysis. The pH values ranging from 3 to 6.5 were evaluated in 0.1 M PBS buffer solution (Supporting information, Figure S4). It can be seen that the optimum response pH for 4-BPA was 3, and the response gradually decreased as the pH increased (Figure 4d). This phenomenon could be due to the conductivity loss and the presence of carboxyl groups with the increase in pH [44].

**Figure 5:**
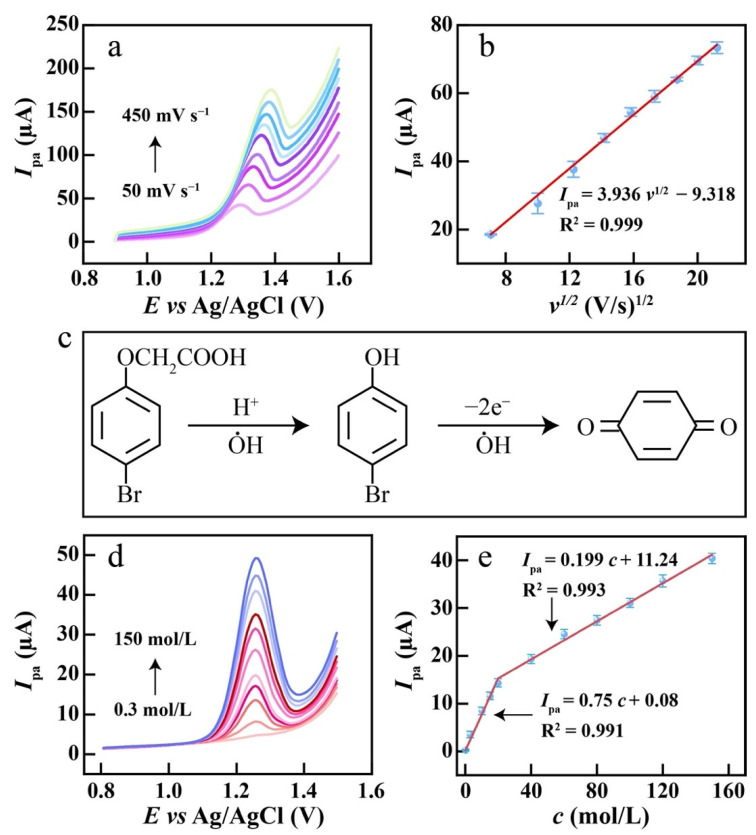
(**a**) LSVs of CeO_2_/eGr/GCE in 0.1 M PBS solution containing 20 µmol L^−1^ 4-BPA at 50, 100, 150, 200, 250, 300, 350, 400, 450 mV s^−1^, (**b**) the plot of *I*_pa_ vs. *v*^1/2^, (**c**) the proposed electrochemical reaction process of 4-BPA, (**d**) DPVs of CeO_2_/eGr/GCE in 0.1 M PBS solution containing 0.3, 3, 10, 15, 20, 40, 60, 80, 100, 120, 150 µmol L^−1^ 4-BPA, (**e**) the plot of the oxidation peak current vs. the concentration of 4-BPA.

The oxidation process of 4-BPA on CeO_2_/eGr/GCE was further studied by linear sweep voltammogram (LSV); different scan rates (50 mV s^−1^ to 450 mV s^−1^) were conducted and 20 µmol L^−1^ 4-BPA was used. As exhibited in Figure 5a, the *I*_pa_ increased when the scan rates increased. Moreover, the oxidation peaks positively shifted. More importantly, the oxidation peak current increased linearly with the square root of scan rates (Figure 5b), the linear regression is *I*_pa_ = 3.936*v*^1/2^ − 9.318, R^2^ = 0.999 and the linear relationship for ln(*I*) versus ln(*v*) was established and the slope was found to be 0.82 (Supporting information, Figure S5), which indicates that the electrochemical oxidation of 4-BPA on CeO_2_/eGr/GCE was controlled by a mixed process [45]. The relationship between *E*_pa_ and ln *v* is presented by Laviron’s theory [46]:

(4) $$E_{\mathrm{pa}}=E^{0}+\left(\frac{RT}{\alpha nF}\right)\ln\left(\frac{RTk^{0}}{\alpha nF}\right)+\left(\frac{RT}{\alpha nF}\right)\ln v$$

where α is the charge transfer coefficient, *E*^0^ is the apparent potential, *n* is the number of the electron, *v* is the scan rate, the values of *R*, *T* and *F* are 8.314 J K^−1^ mol^−1^, 298 K and 96485 C mol^−1^, respectively. Therefore, the number of electrons can be calculated via the linear equations of *E*_pa_ − ln *v* (Supporting information, Figure S6). Generally, for an irreversible electrode process, the value of α is assumed to be 0.5. Hence, the value of *n* is calculated to be 2. Therefore, the electrocatalytic oxidation mechanism of 4-BPA is proposed in Figure 5c, where 4-BPA will firstly be degraded to 4-bromophenol. Then, the 4-bromophenol will be electrochemically oxidized to enzoquinone [47]. The whole process has two electrons involved, which is in accordance with the calculated results from Laviron’s theory.

DPV shows the sensitive response to low concentrations as compared to LSV. Therefore, DPV was used to detect 4-BPA in PBS solution with different concentrations. As illustrated in Figure 5d, the peak current increases linearly with the concentrations of 4-BPA varying from 0.3 to 150 µM. However, there are two linear relationships obtained. From Figure 5e, in the range of 0.3 to 20 µM, the linear regression equation is *I*_pa_ = 0.75*c* + 0.08, (R^2^ = 0.991), and from 20 to 150 µM, the linear relationship is *I*_pa_ = 0.199*c* + 11.24, (R^2^ = 0.993). Moreover, the lowest detection limit (LOD) was calculated to be 0.06 µmol L^−1^ according to the following equation of 3 s/m, where m is the slope of the regression equation and s is the standard deviation of the response.

### 3.4. The Repeatability and the Anti-Interference Ability of the CeO_2_/eGr/GCE

**Figure 6:**
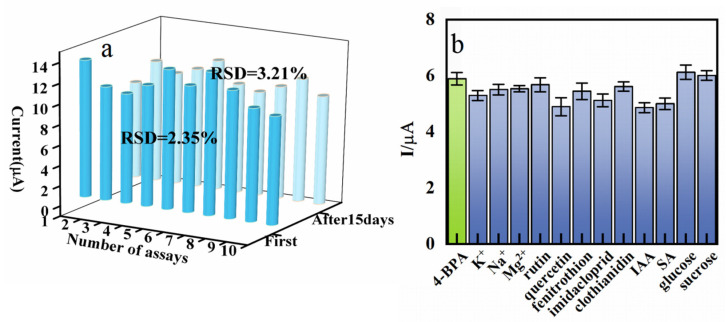
(**a**) The repeatability of current response for the CeO_2_/eGr/GCE in 0.1 M PBS (pH = 3) with 10 successive assays at first day and after 15 days. (**b**) The influence of other compounds on the detection of 4-BPA on CeO_2_/eGr/GCE.

The repeatability of the CeO_2_/eGr/GCE was carried out with 10 µmol L^−1^ 4-BPA by means of LSV (Supporting information, Figure S7). After 10 continuous measurements (Figure 6a), the relative standard deviation (RSD) of the oxidation peak currents was found to be 2.35% for 4-BPA. After storing the electrode at 4 °C for 15 days, the electroactive oxidation currents of 4-BPA reduced 3.21% compared to the original value. These results indicate that the proposed sensor has good stability and repeatability.

To estimate the anti-interference ability of the CeO_2_/eGr/GCE, some regular interfering species were tested. From Figure 6b, no considerable interferences were observed in the presence of fifty-fold excess K^+^, Na^+^, Mg^2+^, glucose, sucrose, and ten-fold rutin, quercetin, fenitrothion, imidacloprid, clothianidin, IAA and SA (peak current change < 6%).

### 3.5. The Practical Application of the CeO_2_/eGr/GCE

To evaluate the practicability of CeO_2_/eGr/GCE, the sensor was used to detect 4-BPA in real apple samples; the analytical results are listed in Table 1. No response of 4-BPA was found in the apple sample, and the recoveries were evaluated by the standard addition method. The recoveries are in the range of 90–108%. This proposed CeO_2_/eGr sensor shows great potential for the detection of plant growth regulators in the agricultural industry.

## 4. Conclusions

In this work, we used an eco-friendly method of electrochemical exfoliation to prepare eGr and the hydrothermal method to prepare CeO_2_. Then, we constructed a selective, sensitive electrochemical method based on a eGr/CeO_2_ composite and modified GCE to electrochemically detect 4-BPA. The prepared CeO_2_/eGr sensor exhibited excellent electrocatalytic activity due to the synergistic effect of the excellent catalytic active sites of CeO_2_ and good electron transference of the eGr. The developed CeO_2_/eGr sensor has an active surface area of 0.097 cm^2^ and a roughness factor of 2.425. The optimized ratio of CeO_2_:eGr is 1:1 for 4-BPA determination. The CeO_2_/eGr sensor exhibited good linearity in a wide range from 0.3 to 150 μmol/L and the lowest detection limit of 0.06 μmol/L for 4-BPA detection. Electrochemical oxidation of 4-BPA followed a mix-controlled process, which involves 2e in the transference processes. In addition, there were no significant interfering substances among K^+^, Na^+^, Mg^2+^, rutin, quercetin, fenitrothion, imidacloprid, clothianidin, IAA, SA, glucose, and sucrose. The proposed electrochemical sensor showed excellent repeatability with the RSD of 2.35% for 10 measurements. In addition, the recoveries of the proposed CeO_2_/eGr sensor were evaluated by the standard addition method, and are in the range of 90–108%. The low cost and easily-made sensor has great potential for detecting other plant growth regulators.

## Figures and Tables

**Table 1:** Results of the recovery analysis of 4-BPA in apple sample (*n* = 3).

Sample	Added Value (μM)	Determined Value (μM)	Recovery (%)
1	0	Not detected (a, b)	-
2	0.5	0.45 ± 0.01	90%
3	1	1.08 ± 0.04	108%
4	3	2.78 ± 0.03	93%
5	5	4.86 ± 0.04	97%

a The 4-BPA level determined by the proposed CeO_2_/eGr/GCE. b The 4-BPA level determined by the HPLC system.

## Data Availability

Not applicable.

## Supplementary Materials

The following supporting information can be downloaded at: https://www.mdpi.com/article/10.3390/bios12090760/s1, Figure S1: the plot of peak currents vs. *v*^1/2^ for (a) CeO_2_/GCE, (b) GCE, (C) eGr/GCE in the presence of 5 mM [Fe(CN)_6_]^3−/4−^ solution in aqueous of 0.1 M KCl. Figure S2: LSVs response of 10 µM 4-BAP to different volumes of composite in PBS solution (pH = 3). Figure S3: LSVs for 4-BPA detection in different buffer solution. Figure S4: Influence of pH on 4-BPA detection in 0.1 M PBS solution. Figure S5: the plots of ln *I* vs. ln *v* for 20 µM 4-BPA at 0.05, 0.1, 0.15,0.2, 0.25, 0.3, 0.35, 0.4, 0.45 V s^−1^. Figure S6: the plots of *E*_pa_ vs. ln *v* for 20 µM 4-BPA at 0.05, 0.1, 0.15,0.2, 0.25, 0.3, 0.35, 0.4, 0.45 V s^−1^. Figure S7: (a) The LSVs of the CeO_2_/eGr/GCE in 0.1 M PBS (pH = 3) at first day and after 15 days. (b) 10 continuous measurements of 4-BPA on CeO_2_/eGr/GCE.
