# Supplementary material for: Electrochemical Determination of 4-Bromophenoxyacetic Acid Based on CeO2/eGr Composite
Source: Biosensors (Basel). 2022 Sep 15;12(9):760. doi: 10.3390/bios12090760 (PMC9496571; doi:10.3390/bios12090760)
Supplement: Supplementary file 1 [file biosensors-12-00760-s001.zip › biosensors-1915523-supplementary.pdf]

Electrochemical Determination of 4-Bromophenoxyacetic Acid Based on eGr/CeO_2 Composite

Haijun Du [1,†], Yan Zhang [1,†], Xin Wang [1,2], Huali Hu [1], Jixing Ai [1], Huanxi Zhou [2,3], Xia Yan [3], Yang Yang [2,*] and Zhiwei Lu [4,*]

1 School of Chemical Engineering, Guizhou Minzu University, Guiyang 550025, China
2 School of Materials Science and Engineering, Guizhou Minzu University, Guiyang 550025, China
3 School of Mechatronics Engineering, Guizhou Minzu University, Guiyang 550025, China
4 College of Science, Sichuan Agricultural University, Ya'an 625014, China
* Correspondence: y.yang@gzmu.edu.cn (Y.Y.); zhiweilu@sicau.edu.cn (Z.L.)
† These authors contributed equally to this work.

Supplementary materials

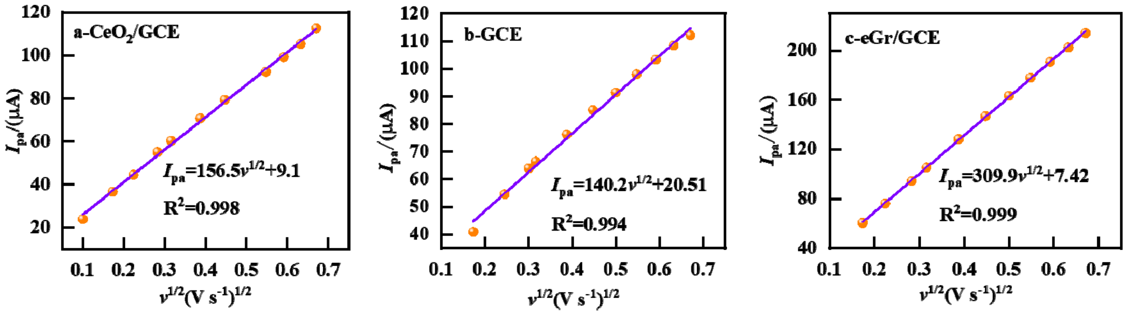

Figure S1. The plot of peak currents vs. $v^{1/2}$ for (a) CeO_2/GCE, (b) GCE, (c) eGr/GCE in the presence of 5 mM $[Fe(CN)_6]^{3-/4-}$ solution in aqueous of 0.1 M KCl.

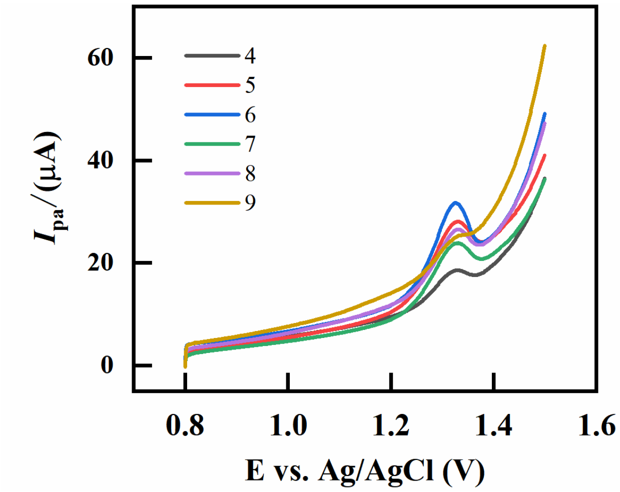

Figure S2. LSVs response of 10 μM 4-BAP to different volumes of composite in PBS solution (pH=3).

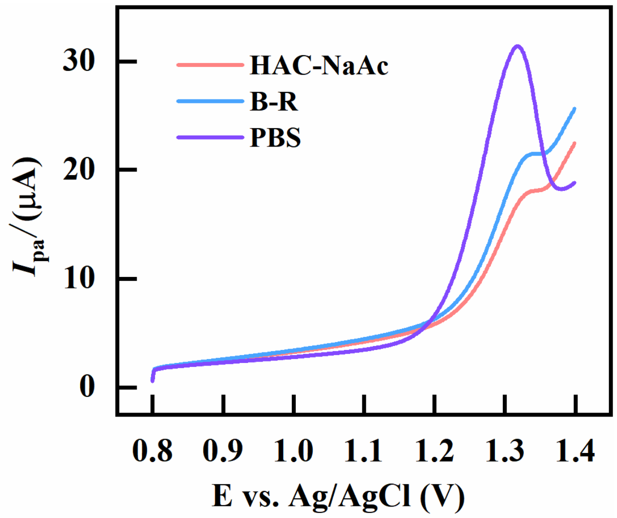

Figure S3. LSVs for 4-BPA detection in different buffer solutions.

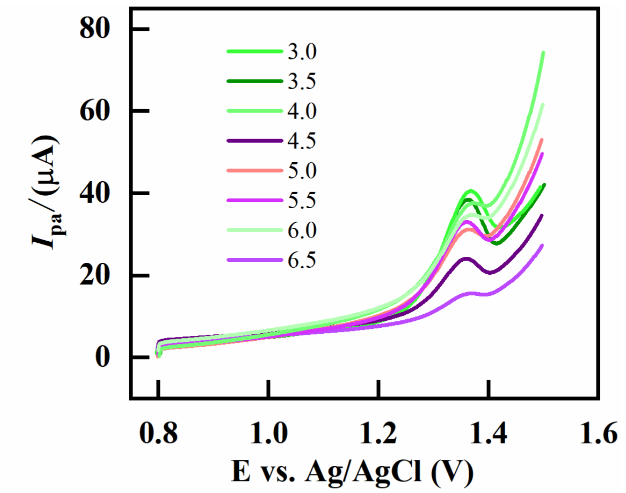

Figure S4. Influence of pH on 4-BPA detection in 0.1 M PBS solution.

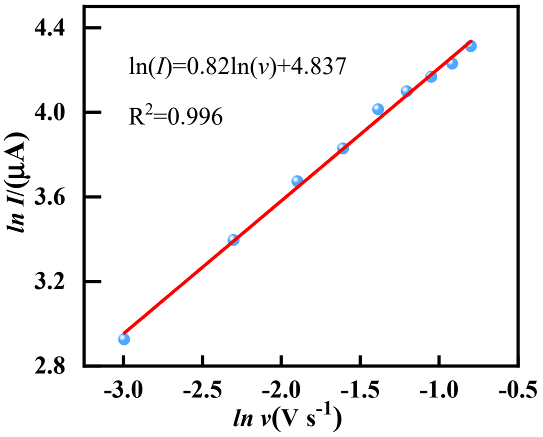

Figure S5. The plots of ln *I* vs. ln *v* for 20 μM 4-BPA at 0.05, 0.1, 0.15,0.2, 0.25, 0.3, 0.35, 0.4, 0.45 V s^{-1}.

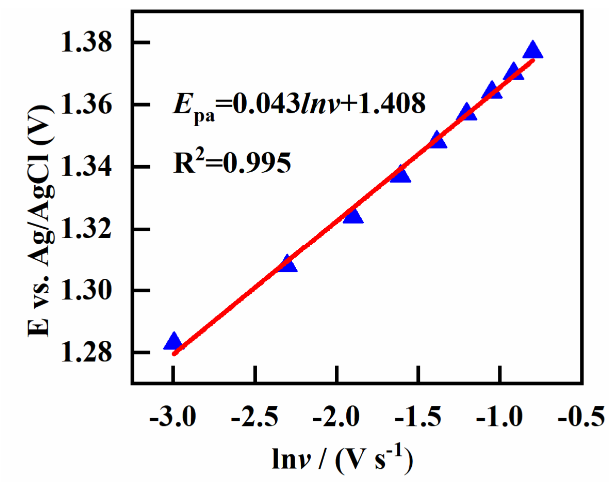

Figure S6. The plots of E_{pa} vs. ln v for 20 μM 4-BPA at 0.05, 0.1, 0.15,0.2, 0.25, 0.3, 0.35, 0.4, 0.45 V s^{-1}.

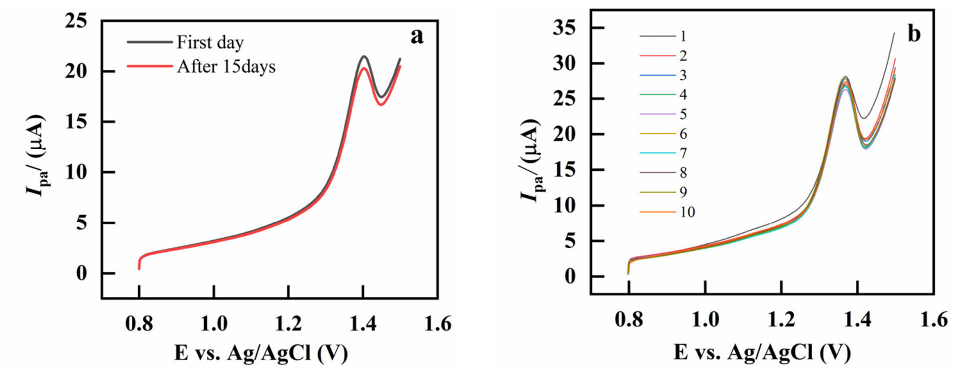

Figure S7. (a) The LSVs of the CeO_2/eGr/GCE in 0.1 M PBS (pH = 3) at first day and after 15 days. (b) 10 continuous measurements of 4-BPA on CeO_2/eGr/GCE.
